# State-of-the-Art Functional Biomaterials in China

**DOI:** 10.3390/jfb15010023

**Published:** 2024-01-15

**Authors:** Yuqin Qiao, Huiliang Cao

**Affiliations:** 1 School of Life Sciences, Shanghai University, Shanghai 200444, China; 2 Interfacial Electrochemistry and Biomaterials, School of Materials Science and Engineering, East China University of Science and Technology, Shanghai 200237, China; 3 Engineering Research Center for Biomedical Materials of Ministry of Education, East China University of Science and Technology, Shanghai 200237, China; 4 Key Laboratory for Ultrafine Materials of Ministry of Education, East China University of Science and Technology, Shanghai 200237, China

## 1. Introduction

In recent years, rapid advancements in multidisciplinary fields (materials, biology, chemical physics, etc.) as well as emerging techniques in biomedical sciences and materials processing have led to a substantial evolution of biomaterials. Distinct from traditional biomedical materials, which are designed to adapt to their microenvironments for tissue integration, functional biomaterials are expected to dynamically and actively instruct cell responses to promote tissue regeneration and tackle biomedical engineering challenges [1]. In China, functional biomaterial-based therapeutics has been identified as one of the main strategies to meet public health problems nationally. Thus, besides pursuing the innovation of functional biomaterials, accelerating their journey from the bench to clinical practice is a top priority. Academic researchers, clinical professionals, and industry partners have been encouraged to collaborate to achieve this goal and improve clinical outcomes.

This Special Issue, “The State-of-the-Art Functional Biomaterials in China”, presents 13 research and review articles collecting the latest research findings and developments. The contributed manuscripts mainly focus on providing solutions to the challenges and limitations faced by current implantable biomedical devices, which can be divided into three categories: strategies to treat implant-associated infections, multifunctional bioactive materials for tissue engineering, and fabrication techniques.

## 2. Overview of Published Articles

Bacterial infection is a common complication associated with various biomaterial applications and has conventionally been treated by the administration of antibiotics [2]. However, the overuse and misuse of antibiotics have led to increasing antibiotic resistance, which poses a serious public health threat around the world [3]. Thus, particular attention is being given to alternative antibacterial agents or biomaterials-based delivery systems that can improve the uptake of antibiotics at specific targeted sites. Yang and co-workers (contribution 1) report the strong antimicrobial effects of silver-doped Zr implant abutments against oral microorganisms, including *P. gingivalis* and *S. mutants*. From a clinical point of view, this functionalized abutment demonstrates good biocompatibility with human gingival fibroblasts because of the stability and low leaching property of silver nanoparticles. Ye et al. (contribution 2) describe how ZnO nanoparticles maximize their antimicrobial activities by improving the bacterial phagocytosis efficiency of polymorphonuclear neutrophils. These synergized effects promote the host’s resistance to pathogen invasion and alleviate subcutaneous immune cell infiltration in vivo. Besides inorganic antimicrobial agents, this delivery system offers a way to increase the effectiveness of antibiotics and is considered another promising strategy to overcome antimicrobial resistance. Li et al. (contribution 3) designed a multi-stimuli-responsive multilayer coating with controlled release of chlorhexidine acetate (CHA) to address bacterial infections that occur when medical devices (such as urinary catheters or peripheral venous catheters) are connected to open wounds in humans. The coating’s multi-responsive abilities, high bactericidal capability (>99% against *E. coli* and *S. aureus*) and reusability (six times), as well as its ease of adoption onto different substrates, are the key advantages it offers in real clinical scenarios. The implantation site is a complex and dynamic microenvironment that involves a sequence of molecular, cellular, and physiological events. Generally, host immune cells (macrophages, neutrophils, T cells, etc.) are well recognized to have a major impact on the outcome of bacterial infection [4]. However, the responses of bacteria to other mammalian cells remain largely unknown. Inspired by the theory of “the race for the surface”, Chen and co-workers (contribution 4) found that tantalum surfaces exhibited a lower infectious state than titanium in an implant-related tibia osteomyelitis model, despite not revealing any antibacterial activity against *S. aureus* in vitro. Bacteria preferentially adhere to the surfaces of mammalian cells rather than those of materials, which may be detached from dead-cell-containing surfaces to reduce the production of biofilms and the occurrence of implant infection. Chen et al.’s study brings a new perspective to the manufacture of implantable materials with antimicrobial activities. In another research study, Zhi and co-workers (contribution 5) demonstrate that calcium- and protein-conditioned titanium may act against bacterial colonization (*S. mutants* and *P. gingivalis*) by releasing calcium ions and creating a basic local pH microenvironment to regulate the conformation of fibrinogen. Their study solidly confirms the indirect antibacterial strategy originally proposed by Cao et al. and provides a promising approach to tackle peri-implant infections [5].

In the field of tissue engineering, antibacterial activity has become an essential requirement for diverse biomaterials [6]. Peng et al. (contribution 6) review the preparation methods of hydrogels attached to substrate surfaces, assess the advantages and limitations of each method when introducing antibacterial substances, as well as evaluate three major antibacterial strategies applied in hydrogels, namely bacterial repellency and inhibition, contact surface killing of bacteria, and release of antibacterial agents. They also discuss the current challenges for researchers in this field, such as chemical stability and sterilization, which may have long-term effects on the human body. In another review article, Zhang and co-workers (contribution 7) discuss recent progress in metal–organic frameworks (MOFs) as antibacterial agents and illustrate their possible molecular mechanisms, such as physical interaction, component release, chemical dynamic therapy (CDT), photodynamic therapy (PDT), photothermal therapy (PTT), sonodynamic therapy (SDT), and synergistic therapy. The authors also raise concerns about these frameworks’ future clinical use. For example, given that MOFs are commonly composed of metal centers and organic ligands, more studies are still needed on the effects of MOF degradation products on human metabolism. Notably, not only can biomaterials be used as antibacterial agents, but they also allow for a targeted and controlled release of antibacterial agents to increase efficiency and safety. Yang and co-workers (contribution 8) summarize recent advances in bacteria-responsive drug delivery systems (DDSs) used for combating bacterial infections, categorizing them by trigger mode, including physical-stimuli-responsive, virulence-factor-responsive, host-immune-response responsive, and their combinations. Although DDSs may suffer from limited specificity and accuracy, the pattern of on-demand drug release and the implementation of multiple functions for tissue repair/regeneration make them one of the most important aspects of biomedical devices. Clinically, the available approaches and strategies to combat bacterial infections are also highly dependent on tissue microenvironment and on the types of biomaterials employed. Cao and co-workers (contribution 9) give a brief overview of antibacterial designs for implantable medical devices from the perspectives of unpredictable onset and site-specific incidence, possibly involving multiple and resistant pathogenic strains. In their paper, Cao et al. intend to illustrate the complex relationships of these devices and figure out future directions for promoting clinical translations.

The ultimate goal of functional biomaterials is to stimulate tissue repair/regeneration following injury and damage [1]. When exposed to functional biomaterials, cells can sense signals from the material’s features, such as its topography and chemical composition, and convert them into biological responses [7,8]. Zhang et al. (contribution 10) fabricated four different submicron-grooved polystyrene films using soft lithography. The results show that submicron groove structures can significantly transform the morphology and cytoskeleton of Schwann cells and upregulate the gene expression of Schwann cells responsible for axonal regeneration and myelination, which is promising for peripheral nerve repair. Xie and co-workers (contribution 11) fabricated a duplex film with an inner MnOOH layer and an outer FeOOH layer on PEO-treated AZ31 using a simple immersion processes. The oxyhydroxide film completely sealed the pores on the PEO surface, thus providing better corrosion resistance. The modified sample exhibited improved osteogenic activity in vitro and enhanced bone regeneration in vivo, indicating promising potential for orthopedic applications. Wang and co-workers (contribution 12) investigated the mechanism of magnesium in regulating osteogenic activity. The results reveal that the AMPK/mTOR signaling pathway is involved in the process of autophagy associated with magnesium-induced osteogenic differentiation of BMSCs. Their study advances our understanding of the link between autophagy induction and osteogenic differentiation. Recently, much evidence has suggested that tissue repair/regeneration is not simply regulated by local signals and does not occur independently of other organ functions; instead, it requires the precise coordination of different organ systems [9]. Zhao et al. (contribution 13) provide an overview of biomaterials-mediated immune responses that regulate bone regeneration, methods to assess the bone immunomodulatory properties of biomaterials, as well as the strategies that can be used for future bone tissue engineering applications.

The development of functional biomaterials is also highly reliant on the fine-tuning of material structures and properties, as well as on progress in fabrication techniques [10]. Surface modification has been well recognized as an economical and efficient approach to endow the surfaces of conventional biomaterials with bioactivity and multifunctionality for therapeutic purposes [11]. This can help retain the favorable bulk properties that determine whether a material can adapt to the human body. For example, plasma immersion ion implantation and deposition (PIII&D) can introduce metal or gas elements to objects with flexible shapes [12]. Typically, the depth of the modified layer is relatively shallow (<100 nm), and the amount of the doped element is relatively low. Thus, Ag-doped zirconia implant can exhibit good biocompatibility with human gingival fibroblasts (contribution 1). Among various surface modification techniques, plasma electrolyte oxidation (PEO) is extensively used to produce porous and thick metal oxide coatings on metals (e.g., titanium, magnesium, aluminum, and their alloys) in industrial and biomedical applications, especially in dental implants, bone fusion, bone fixation, etc. [13]. It is for this reason that this technique has been widely explored by researchers and used to develop a variety of functional biomaterials (contributions 11 and 12). As a high-throughput tool for surface patterning, soft lithography can create ordered topographical features (grooves, pillars, and pores) and benefits from ease of preparation, high efficiency, and reusability [14]. It plays an important role in exploring the relationship between surface topography and cellular behaviors (contribution 10). Distinct from surface modification, nanomaterials such as nanoparticles and MOFs are usually synthesized by one-pot, hydrothermal, ultrasonic-assisted, electrochemical, and mechanochemical methods [15] (contributions 7 and 8). The high cost of nanomaterials, as well as complications in their purification and stability, are the main factors influencing their future applications.

## 3. Conclusions

In summary, this Special Issue, entitled “State-of-the-Art Functional Biomaterials in China”, presents a diverse collection of research papers and review articles on trailblazing antibacterial surfaces, advanced multifunctional biomaterials, and clinical studies on the applications of biomaterials. These collaborations of academic and clinical research will provide unique and essential insights into functional biomaterials and increase the efficiency of their clinical translation.

## Data Availability

Not applicable.
